# Novel Strategies and Therapeutic Advances for Bladder Cancer

**DOI:** 10.3390/cancers17132070

**Published:** 2025-06-20

**Authors:** Matthew I. Ehrlich, Robert D. Fox, Karie D. Runcie, Mark N. Stein, Alexander Z. Wei

**Affiliations:** Division of Hematology & Oncology, Columbia University Irving Medical Center, New York, NY 10032, USA; me2771@cumc.columbia.edu (M.I.E.); rf2763@cumc.columbia.edu (R.D.F.); kr2836@cumc.columbia.edu (K.D.R.); mns2146@cumc.columbia.edu (M.N.S.)

**Keywords:** bladder cancer, urothelial carcinoma, novel therapies, bladder-sparing, trimodality therapy

## Abstract

**Background/Objectives:** To summarize the relevant trials relating to novel strategies and therapeutic advances in the treatment of bladder cancer. **Methods:** A comprehensive review of the literature and recent/ongoing clinical trials was conducted, focusing on novel treatments and strategies for bladder cancer. Trials started or published as of 2020 were included. **Results:** The standard of care for MIBC remains neoadjuvant chemotherapy with perioperative immunotherapy in the cisplatin-eligible population, while guidelines do not exist for cisplatin-ineligible patients. Strategies under investigation include combinations of chemotherapy, immunotherapy, radiation, and/or novel therapies, such as ADCs, targeted agents, intravesical treatments, and personalized vaccines. Bladder-sparing approaches using these novel therapies are also being studied. In the advanced/metastatic setting, enfortumab vedotin plus pembrolizumab has supplanted platinum-based chemotherapy as the first-line treatment option. For those with contraindications, or who progress, strategies under investigation include newer immunotherapies and ADCs, novel small molecule inhibitors, and cellular therapies. **Conclusions:** The treatment landscape for bladder cancer has changed drastically within the last few years. Ongoing trials hope to build on this success by investigating bladder-sparing strategies for MIBC and novel systemic therapies in advanced patients.

## Simple Summary

Platinum-based chemotherapy had been the backbone of bladder cancer treatment for decades until the advances of the last few years. Chemoimmunotherapy regimens and antibody–drug conjugates have revolutionized the treatment of both localized and advanced disease, leading to changes in the consensus guidelines. Ongoing trials are investigating bladder-sparing approaches in the localized setting, as well as novel therapies for all stages of disease.

## 1. Introduction

Bladder cancer ranks as the ninth most common cancer by incidence worldwide, with over 600,000 new cases diagnosed per year as of the latest statistics [1]. Under current projections, the yearly incidence rate is predicted to reach nearly one million cases by 2040 [2]. This disease is also quite morbid, accounting for more than 200,000 deaths per year and rising [3]. Bladder cancer is typically a disease of older age, with an average age at diagnosis of 73, and is associated with risk factors such as tobacco use, various carcinogens, and chronic inflammation of the urinary tract, as well as familial predispositions including mutations in DNA repair genes, such as MSH2 and MLH1, and cellular metabolism genes, such as IDH1 and ME1 [4,5].

Approximately 90% of all bladder cancer diagnoses are urothelial carcinoma (UC), with other histological variants, including squamous cell carcinoma, adenocarcinoma, and small cell carcinoma, comprising the remaining 10% [6]. Bladder cancer can be further stratified into non-muscle invasive (NIMBC), confined to the mucosa or at most invading into the lamina propria, and muscle invasive (MIBC), which penetrates through the muscularis into deeper layers of the bladder wall [7].

The standard treatment for localized MIBC involves a multimodal approach consisting of chemotherapy and radical cystectomy; however, despite definitive treatment, many patients do recur, typically within the first two years [8]. Primary advanced or metastatic disease also confers a poor prognosis, with five-year relative survival rates of 40% and 9%, respectively [9]. As such, better treatment options are desperately needed.

This review provides a brief overview of the current standard-of care in invasive bladder cancer followed by a comprehensive discussion of recently completed and ongoing clinical trials utilizing novel treatments and strategies for patients with muscle-invasive and advanced UC. Emerging therapies are diverse, including immunotherapies, antibody–drug conjugates, cellular therapies, and targeted agents. Innovative bladder-sparing strategies are also on the horizon. We examine these recent advancements and outline the evolving landscape of bladder cancer treatment.

## 2. New Strategies for Muscle-Invasive Bladder Cancer

The standard-of-care treatment for muscle-invasive bladder cancer (MIBC) has historically consisted of neoadjuvant cisplatin-based chemotherapy followed by radical cystectomy (RC). Several studies have demonstrated the benefit of neoadjuvant treatment with dose-dense methotrexate, vinblastine, doxorubicin and cisplatin (ddMVAC), or gemcitabine plus cisplatin (GC) [10,11,12,13]. These cisplatin-based regimens have remained the recommendations from major cancer societies for over thirty years until the recent addition of durvalumab to chemotherapy.

For patients who are not cisplatin-eligible or surgical candidates, trimodality therapy (TMT) consisting of chemoradiation and surgery can be considered. As modern systemic therapeutic options have become more effective, one question asked by both clinicians and patients is whether radical cystectomy is still required for all patients with MIBC. Studies attempting to answer this question will be discussed.

Clearly, within the treatment schema for MIBC, cisplatin eligibility has classically dictated treatment options. We will discuss the literature as it relates to this structure and note that the advent of novel therapies is likely to change this paradigm moving forward (Table 1).

### 2.1. Perioperative Chemoimmunotherapy with Radical Cystectomy

The latest major change to consensus management of MIBC comes from the results of the NIAGARA trial [14]. This phase III study randomized cisplatin-eligible patients to receive neoadjuvant GC with or without perioperative durvalumab (PD-L1 inhibitor). Five hundred and thirty patients were included in the intention-to-treat analysis. Estimated event-free survival (EFS) at 24 months was 67.8% in the durvalumab arm compared to 59.8% in the control arm (HR 0.68, *p* < 0.0001). The second primary endpoint of pathologic complete response (pCR) reported in a re-analysis in April 2024 revealed rates of 37.3% in the durvalumab arm compared to 27.5% in the comparator arm (OR 1.6, *p* = 0.0005). The authors also published overall survival (OS) data after a median follow-up of 46.3 months; the 24-month survival was 82.2% in the durvalumab group compared to 75.2% in the control group (HR 0.75, *p* = 0.016). Of note, this trial also allowed for a wider range of cisplatin-eligible patients by lowering the creatinine clearance cutoff to 40 mL/min, which may have broader implications in the bladder cancer space. Additionally, a recent exploratory analysis of the NIAGARA trial examined the association of circulating tumor DNA (ctDNA) with clinical outcomes [15]. Overall, the ctDNA positivity rate was 57% at diagnosis, decreasing to 22% after neoadjuvant treatment; clearance rates were 41% and 31% in the durvalumab and comparator arms, respectively. Among the cohort who had positive ctDNA assays prior to RC, 97% did not have a pCR at time of surgery.

Similarly, KEYNOTE-866 (NCT03924856) is ongoing, investigating GC with pembrolizumab [16], while the ENERGIZE trial (NCT03661320), described further below, studies GC with nivolumab in addition to the investigational drug linrodostat [17]. There is currently no standard-of-care neoadjuvant regimen for cisplatin-ineligible patients with MIBC. The NURE-COMBO study (NCT04876313) attempted to remedy this by using a combination of chemoimmunotherapy [18]. In this phase II study, 31 cisplatin-ineligible patients received neoadjuvant nab-paclitaxel and perioperative nivolumab. As of the first results report in September 2024, 32.3% of patients achieved a pCR, and including those who underwent repeat transurethral resection of bladder tumor (TURBT), 70.9% achieved pathologic downstaging. The 12-month EFS was 89.8%. Using a different strategy, the RAD-VACCINE MIBC trial (NCT05241340) combines checkpoint inhibitors and stereotactic body radiation therapy (SBRT) [19]. In this phase II single-center study, cisplatin-ineligible patients receive neoadjuvant sasanlimab (PD-1 inhibitor) with concurrent SBRT followed by RC.

While traditional management of localized disease involves neoadjuvant chemotherapy, there is evidence to support adjuvant treatment if chemotherapy is not given in the neoadjuvant setting or if the patient is cisplatin-ineligible—particularly for pathologic T3, T4, or node-positive disease [20,21]. CHECKMATE-274 demonstrated a survival benefit with adjuvant immunotherapy in this population [22]. In this phase III trial, patients with high-risk MIBC were randomized to either adjuvant nivolumab or placebo after RC. High-risk was defined as pT3-T4a or node-positive in patients who did not receive neoadjuvant treatment and declined or were not eligible for adjuvant cisplatin-based chemotherapy, or ypT2-T4a or node-positive for those who received neoadjuvant cisplatin-based chemotherapy. The median disease-free survival (DFS) was 20.8 months in the nivolumab group compared to 10.8 months in the placebo group, and the six-month disease-free rate was 74.9% vs. 60.3%, favoring the nivolumab arm (HR 0.70, *p* < 0.001). This benefit was observed regardless of nodal status, PD-L1 status, or use of neoadjuvant chemotherapy. An update provided in 2024 reported an overall survival benefit with nivolumab, 69.5 months compared to 50.1 months (HR 0.76). Median OS was not yet reached in either arm (HR 0.56) among patients with PD-L1 status ≥ 1% [23]. The currently enrolling MODERN trial (NCT05987241) is investigating adjuvant immunotherapy combinations based on circulating tumor DNA (ctDNA) status. This phase II/III study enrolls patients who have undergone radical cystectomy and are at high risk of recurrence—for those not receiving chemotherapy, pT3-4, or pN+ on cystectomy and ineligible for or refusing adjuvant cisplatin-based chemotherapy, or for those who received neoadjuvant cisplatin-based chemotherapy, ypT2-4, or ypN+ on cystectomy. Patients will then have a commercial ctDNA assay performed and will be randomized into one of two arms based on their status. Those with a positive ctDNA assay will either receive adjuvant nivolumab plus relatlimab (LAG-3 inhibitor) or nivolumab alone. Those with a negative ctDNA assay will be randomized to either adjuvant nivolumab or active surveillance (in which case if ctDNA turns positive, they will receive nivolumab). Finally, it is relevant to mention the phase III IMvigor010 trial, an early negative study in this space concerning the use of adjuvant atezolizumab [24]. An updated survival analysis stratified by ctDNA status revealed a trend towards benefit of adjuvant therapy versus observation in those with ctDNA positivity. Patients with ctDNA positivity showed evidence of longer OS with atezolizumab compared to observation (29.8 vs. 14.1 months; HR 0.59), while those with ctDNA negativity had similar survival among the two arms [25]. Building on these results, the ongoing IMvigor011 trial (NCT04660344) is a double-blind, randomized phase III study directly assessing the benefit of adjuvant atezolizumab versus placebo in patients with MIBC who have ctDNA positivity post-cystectomy [26].

### 2.2. Neoadjuvant Antibody–Drug Conjugates (ADC) with Radical Cystectomy

Within the past year, there has been a paradigm shift in the treatment of advanced/metastatic urothelial cancer with the approval of enfortumab vedotin (EV), an antibody–drug conjugate targeting nectin-4, in combination with pembrolizumab, as demonstrated in EV-302 [27]. Concomitantly, EV is also being explored in the localized MIBC setting. In the multi-arm EV-103 study (NCT03288545), which focuses on the advanced/metastatic setting, several cohorts include cisplatin-ineligible patients with MIBC. Cohort H is a phase Ib/II study of neoadjuvant EV in cisplatin-ineligible patients [28]. Twenty-two patients were enrolled and planned to receive three cycles of EV prior to RC. A pCR rate of 36.4% and a 50% rate of pathological downstaging was observed. In the latest update from May 2024, the EFS rate at 24 months was 62%, while median EFS has not yet been reached [29]. Cohort L investigated perioperative EV in a similar cohort. In this study, 51 patients underwent three cycles of neoadjuvant EV followed by RC, with a plan for six additional cycles of adjuvant EV therapy. The study reported a pCR rate of 34% and pathologic downstaging rate of 42%; final data after adjuvant therapy is still pending [30]. Due to these results, the phase III EV-303 trial was initiated (NCT03924895) [31]. This is a three-arm study in which cisplatin-ineligible patients are randomized to RC plus either perioperative pembrolizumab, perioperative EV plus pembrolizumab, or observation. The phase III VOLGA trial (NCT04960709) similarly investigates perioperative EV with or without intensified immunotherapy in cisplatin-ineligible patients [32]. Patients are randomized to one of three arms: neoadjuvant EV plus perioperative durvalumab plus tremelimumab (CTLA-4 inhibitor); neoadjuvant EV plus perioperative durvalumab; or cystectomy with or without adjuvant nivolumab (in high-risk patients). Additionally, the actively recruiting STAR-EV trial (NCT06394570) is evaluating the combination of EV with SBRT [33]. In this single-center phase I/II study, cisplatin-ineligible patients receive neoadjuvant EV with either sequential or concurrent SBRT in five fractions prior to RC. Due to promising results in the cisplatin-ineligible setting, EV has also been moved to the cisplatin-eligible population. In EV-304 (NCT04700124), cisplatin-eligible patients are randomized to either perioperative EV plus pembrolizumab or neoadjuvant GC in addition to RC [34].

Sacituzumab govitecan (SG), an ADC-targeting trophoblast cell surface antigen 2 (Trop-2), represents another significant, yet controversial, drug in the treatment of urothelial cancer. Initially approved in the metastatic setting after platinum chemotherapy and immunotherapy based on the phase II TROPHY-U-01 trial, sacituzumab govitecan was later withdrawn based on the phase III TROPiCS-04 trial in which it did not meet its OS endpoint and also led to an excess of neutropenic deaths [35]. However, the drug is currently being evaluated for perioperative use in localized MIBC in the multi-cohort phase II SURE study in which cisplatin-ineligible patients receive neoadjuvant SG (SURE-01; NCT05226117) or neoadjuvant SG with perioperative pembrolizumab (SURE-02; NCT05535218) [36]. The study also allows for a bladder-sparing approach (further details below). Results have only been reported for the SURE-01 cohort at this time, with the latest update released in December 2024 [37]. Twenty-five patients were enrolled, of which 21 proceeded to surgery (13 RC, 8 repeat TURBT). Complete clinical response was noted in 44%, and pCR in 48%. After a median follow-up of 22.3 months, the six-month EFS was 80%. Of note, grade ≥ 3 adverse events occurred in 36% of patients, most frequently neutropenia and diarrhea, and two grade 5 events were reported.

Antibody–drug conjugates targeting HER2 represent another promising area of drug discovery. In the phase II RC48-C017 trial (NCT05297552), disitamab vedotin (DV) is under investigation along with the PD-1 inhibitor toripalimab. As of the latest update from February 2025, 47 cisplatin-ineligible patients with tumors demonstrating HER2 expression ≥ 1+ on immunohistochemistry received neoadjuvant DV plus toripalimab. Of 33 patients who underwent RC, the pCR rate was 63.6%, with an increase to 84.6% in those with HER2 IHC 3+. A higher pCR rate was also noted among PD-L1-positive patients (77.8% vs. 62.5%). The 12-month EFS rate was 89 5% [38]. Common adverse events included alopecia (36.4%), elevated transaminases (AST 31.8%, ALT 25%), rash (22.7%), and peripheral neuropathy (22.%); grade 3–4 events were reported in 15.9% of patients.

### 2.3. Targeted Therapies for Localized MIBC

The ENERGIZE study (NCT03661320) is a phase III trial of chemotherapy in combination with perioperative immunotherapy as well as the IDO1 inhibitor linrodostat [17]. This study assigns cisplatin-eligible patients to one of three arms, all including RC: neoadjuvant GC alone; neoadjuvant GC plus perioperative nivolumab; and GC plus perioperative nivolumab and linrodostat.

Erdafitinib, the small molecule inhibitor of FGFR, is under investigation in the neoadjuvant setting in the phase II SOGUG-NEOWIN study (NCT06511648) [39]. Cisplatin-ineligible patients who test positive for FGFR mutations are assigned to one of two cohorts—neoadjuvant erdafitinib monotherapy or neoadjuvant erdafinitib plus the PD-1 inhibitor cetrelimab.

APL-1202 (nitroxoline) is an oral MetAP2 inhibitor with anti-angiogenic and anti-tumor properties. In the phase II ANTICIPATE trial (NCT04813107), cisplatin-ineligible patients are randomized to either neoadjuvant APL-1202 plus the PD-1 inhibitor tislelizumab (group 1) or tislelizumab alone (group 2) followed by RC. As of the interim analysis from January 2024, 42 patients have enrolled, of which 32 were evaluable after RC (10 refused surgery). Pathologic complete response rates were 39% in group 1 and 21% in group 2, and pathologic downstaging occurred in 44% and 21%, respectively [40].

### 2.4. Intravesical Therapy in Combination with Immunotherapy

TAR-200 is a novel intravesical drug delivery system that continuously releases gemcitabine within the bladder. SunRISe-4 (NCT04919512) is a randomized phase II study of this system in combination with immunotherapy in the neoadjuvant setting. Cisplatin-ineligible patients with residual intravesical tumor volume of ≤3 cm are randomized to either TAR-200 gemcitabine plus cetrelimab (cohort 1) or cetrelimab alone (cohort 2) prior to RC. At the interim analysis in May 2024, a total of 120 patients were treated. The pCR rates were 42% in cohort 1 and 23% in cohort 2, and the rates of pathologic downstaging were 60% and 35%, respectively [41].

GC0070 (cremostigene) is an oncolytic adenovirus genetically engineered with an E2F1 promoter region and a GM-CSF transgene; as such, it selectively replicates only in tumor cells deficient in the Rb pathway. In the phase Ib CORE-002 trial (NCT04610671), cisplatin-ineligible receive neoadjuvant intravesical CG0070 with nivolumab followed by cystectomy. As of the latest update from November 2024, 21 patients were treated, with a pCR rate of 42.1% and 12-month recurrence-free survival (RFS) rate of 70.4% [42].

### 2.5. Vaccine Therapy in Combination with Immunotherapy

The personalized neoantigen vaccine PGV001 was studied in a recently completed phase I pilot study in combination with atezolizumab for patients with UC in both the adjuvant and metastatic settings [43]. In the adjuvant setting, patients were required to have features associated with high risk of metastatic recurrence on the resection specimen defined by one of the following: pathological ypT2-T4 or ypN+ after neoadjuvant chemotherapy, or pT3-T4 or pN+ for those not treated with neoadjuvant chemotherapy and declining or ineligible for cisplatin-based adjuvant chemotherapy. PGV001 was administered for up to ten doses, and atezolizumab was given for up to one year. Four patients were treated in the adjuvant setting, of which three displayed no evidence of metastatic recurrence after a median follow-up of 39.5 months.

V940 (mRNA-4157) is a novel individualized vaccine therapy consisting of an mRNA encoding up to 34 tumor neoantigens, which has shown potential in other tumor types, notably melanoma. INTerpath-005 (NCT06305767) is a phase I/II clinical trial of the V940 vaccine plus pembrolizumab with or without EV [44]. The phase I study is a single-arm cohort of cisplatin-ineligible patients who will receive perioperative V940 with pembrolizumab and EV. The phase II study is a randomized cohort of cisplatin-ineligible patients with high-risk pathologic MIBC (ypT2-4a and/or ypN+ after neoadjuvant chemotherapy, or pT3-4a and/or pN+ without neoadjuvant chemotherapy) who will receive adjuvant pembrolizumab with either V940 or placebo.

Autogene cevumeran is another individualized mRNA-based vaccine therapy consisting of up to 20 tumor neoantigens that has been tested both as monotherapy and in combination with immunotherapy in patients with advanced solid tumors. IMCODE004 (NCT06534983) is a randomized phase II trial that evaluates autogene cevumeran with and without nivolumab in the adjuvant setting. The study includes patients with high-risk MIBC defined as (y)pT3-4 or (y)pN+; patients who have not received neoadjuvant chemotherapy must be ineligible to receive adjuvant cisplatin-based chemotherapy upon enrollment.

### 2.6. Cytokine Therapies

PIVOT IO 009 (NCT04209114) is a phase III clinical trial of perioperative nivolumab plus Bempeg (NKTR-214), an immunomodulatory IL-2 prodrug [45]. In this study, cisplatin-ineligible patients were randomized to three arms: perioperative nivolumab plus BEMPEG; perioperative nivolumab monotherapy; or RC alone. The study has been completed, and results are pending.

## 3. Cystectomy-Sparing Approaches

### 3.1. Systemic Therapy

RC is a major life-changing procedure due to the need for urinary diversion and is associated with a mortality and morbidity risk [46]. Every year, between 2000 and 2500 patients undergo RC in the United States [47]. While this procedure can be curative, it is also highly morbid and associated with a significant risk of postoperative complications [48]. Approximately two-thirds of patients experience a postoperative complication within 90 days of surgery, and up to 5% will die. In addition, there are substantial negative changes in postoperative body image, as well as urinary and sexual function. Several retrospective studies and meta-analyses have reported promising outcomes after definitive treatment with systemic therapy and TURBT alone [49,50,51]. Studies have also demonstrated that when given the choice, patients would rather keep their native bladder than undergo radical cystectomy [52]. As such, prospective strategies evaluating bladder-sparing approaches in patients with MIBC are currently underway and may lead to practice and guideline changing recommendations in the near future.

HCRN GU16-257 was a phase II clinical trial in which patients received neoadjuvant gemcitabine/cisplatin/nivolumab followed by clinical restaging with imaging, cystoscopy with biopsies, and urine cytology [52]. Patients achieving a clinical complete response (cCR) were offered to either proceed with cystectomy or forgo surgery and receive adjuvant nivolumab; patients without a cCR proceeded to cystectomy. The study enrolled 76 patients; 33 (43%) had a cCR, of which 32/33 patients—or 97% of those who achieved cCR—opted to forgo immediate cystectomy and receive maintenance nivolumab. At the time of publication after a median of 30 months of follow-up, eight patients with cCR eventually underwent cystectomy for local recurrence and two developed metastatic disease.

The RETAIN studies followed a similar approach to assess which patients might be able to avoid RC. RETAIN-1 was a single-arm phase II noninferiority trial designed to evaluate a risk-adapted approach for MIBC by identifying mutations in DNA damage repair genes that are associated with pathologic downstaging after neoadjuvant therapy [53]. Patients underwent neoadjuvant chemotherapy with accelerated methotrexate, vinblastine, doxorubicin, and cisplatin (AMVAC); pre-treatment TURBT specimens were sequenced. Patients with at least one mutation and no clinical evidence of disease upon restaging entered active surveillance, while the remainder underwent bladder-directed therapy depending on clinical stage. Of the 77 enrolled patients, 33 had an identified mutation and 25 of those entered active surveillance after neoadjuvant therapy. After a median follow-up of 40 months, the 2-year metastasis-free survival rate was 76% in the active surveillance group and 71.1% in the further treatment group (primary endpoint of noninferiority not met). In the surveillance group, 68% of patients had some recurrence, and 48% were metastasis-free with an intact bladder. The 2-year OS was 88% and 82.2% in the surveillance and treatment groups, respectively. Building on these results, the ongoing RETAIN-2 trial (NCT04506554) employs a similar approach but incorporates neoadjuvant chemoimmunotherapy [54]. In this phase II study, a similar cohort of patients received neoadjuvant AMVAC with nivolumab, and again patients with a detectable mutation and a cCR after therapy underwent active surveillance. In the interim analysis published in February 2025, 80 patients were treated, of which 71 were evaluable. Thirty-one patients (44%) had a mutation of interest, of which 71% had a cCR. Overall, 23 patients entered active surveillance (19 who were also mutation-positive), while 35 proceeded to RC. Among cystectomy patients, 46% had pCR and 63% demonstrated pathologic downstaging. After a median follow-up of 18.4 months, 78% of patients on active surveillance remained metastasis-free with intact bladders. Of note, two patient deaths were reported on study, both after completion of three cycles of chemoimmunotherapy, attributed to treatment-related adverse events.

Alliance A031701 (NCT03609216) follows a similar structure to the RETAIN trials [55]. In this phase II study, patients will undergo pre-treatment genetic sequencing of tumor specimens and then receive GC. Patients who demonstrate a DNA damage response mutation and have <pT1 response on clinical restaging are eligible for bladder-sparing treatment; otherwise, patients will undergo RC or chemoradiation.

NeoSTOP-IT (NCT06571708) is a phase II, open-label, randomized trial with continuous Bayesian toxicity monitoring investigating the addition of anti-LAG-3 therapy to standard of care chemoimmunotherapy with bladder-sparing intent for patients with MIBC [56]. Patients with T2-T3, node-negative MIBC will undergo maximal TURBT followed by GC plus cemiplimab (PD-1 inhibitor) with or without fianlimab (LAG-3 inhibitor). Those who achieve cCR may defer RC and continue maintenance immunotherapy with either cemiplimab and fianlimab or cemiplimab alone, while those without cCR will be recommended to undergo RC. Accrual for this study is underway.

In the previously discussed phase II SURE study, cisplatin-ineligible patients receive neoadjuvant SG (SURE-01; NCT05226117) or neoadjuvant SG with perioperative pembrolizumab (SURE-02; NCT05535218). Patients with a cCR who refuse RC are offered repeat TURBT followed by observation alone if no viable high-grade tumor is found.

Finally, HCRN-GU22-598 (NCT06809140) is an accruing phase II single-arm trial investigating EV plus pembrolizumab as a bladder-sparing approach. Patients with T2-T3, node-negative MIBC will receive three cycles of induction combination therapy followed by restaging. Patients achieving cCR will receive 14 cycles of “maintenance” treatment, consisting of EV plus pembrolizumab for the first six cycles, and pembrolizumab alone for the remainder. Patients with any residual disease will undergo cystectomy.

### 3.2. Trimodality Therapy (TMT)

Trimodality therapy represents an alternative to chemoimmunotherapy followed by cystectomy in the guideline-directed management of bladder cancer [57]. TMT refers to maximal TURBT followed by concurrent chemoradiotherapy (CRT) and carries a category 1 recommendation for localized MIBC in NCCN guidelines. Optimal candidates for this strategy include those tumors that present without moderate or severe hydronephrosis, are without concurrent extensive or multifocal carcinoma in situ, and are less than 6 cm in size [54]. To date, there are no prospective randomized, controlled trials comparing TMT to radical cystectomy. Ongoing trials in this space include novel therapies as part of the TMT approach.

NCT02621151 is a phase II trial investigating the combination of gemcitabine and pembrolizumab with concurrent hypofractionated radiation therapy (RT) as a TMT approach. Patients who decline or are ineligible for RC receive pembrolizumab followed by maximal TURBT and whole-bladder RT with concurrent twice weekly gemcitabine and pembrolizumab. At the latest update in 2023, 54 patients were enrolled, and most (88%) completed all therapy [58]. With a median follow-up of 23 months, 12 patients (22%) had tumor recurrences, while another 4 (7%) had NMIBC recurrences. The 2-year bladder-intact DFS rate was 71%, and OS was 83%. Of note, 11% of patients required salvage cystectomy, and ten patients (18%) died during the study period (three from disease progression, one from toxicity, and six unrelated/unknown).

A related ongoing study (NCT03993249) employs nivolumab in addition to chemoradiation. This phase II trial randomizes patients who underwent maximal TURBT to receive either cisplatin-based CRT alone or with nivolumab. At the interim analysis published in September 2024, 72 patients had enrolled in the study. The 2-year relapse-free survival rates were 37.6% in the CRT group and 59.9% in the CRT plus nivolumab group, and the median OS was 24 months and not reached, respectively [59].

IMMUNOPRESERVE is a phase II clinical trial recently completed by the Spanish Oncology Genitourinary Group (SOGUG) [60]. Patients who were ineligible for cystectomy or wished for bladder preservation underwent initial TURBT then received lead-in dosing of durvalumab and tremelimumab, followed by external-beam RT (EBRT). Thirty-two patients were enrolled, of which 81% had a pCR. After a median follow-up of 27 months, two patients underwent salvage cystectomy. The 2-year rates of bladder-intact DFS, metastasis-free survival, and OS were 65%, 83%, and 84%, respectively.

Building on these studies, two large, randomized phase III trials are in progress evaluating TMT with or without immunotherapy. The INTACT (SWOG/NRG 1806) study (NCT03775265) randomizes patients who have undergone maximal TUBRT to CRT with or without atezolizumab [61]. KEYNOTE-992 (NCT04241185) randomizes a similar cohort of patients to receive CRT with or without pembrolizumab [62]. Both trials completed accrual quickly despite being competing studies and activation during the COVID-19 pandemic. Results from these studies are eagerly awaited.

The RAD-SG study (NCT05833867) takes a related but different approach to TMT with the use of sacituzumab govitecan. This is an ongoing single-arm phase I trial in which patients who have undergone maximal TUBRT receive concurrent SG with adaptive RT [63].

## 4. Novel Therapeutics for Advanced Urothelial Cancer

**Figure 1:**
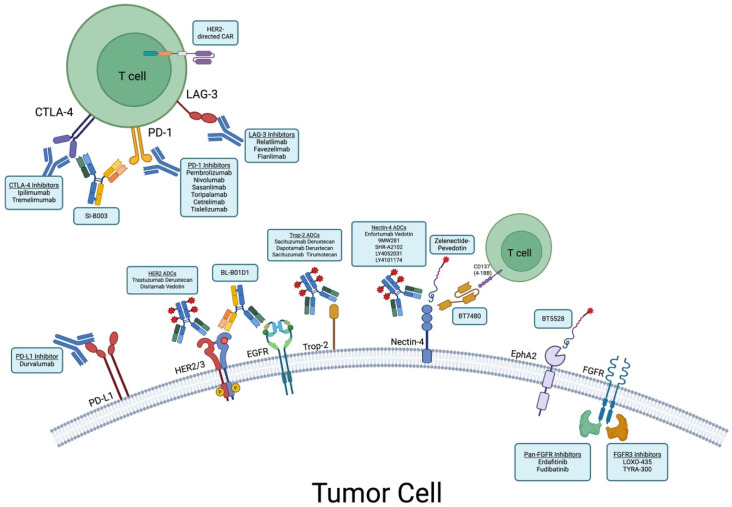
Mechanisms of selected novel therapeutics for the treatment of bladder cancer. Created in BioRender. Ehrlich, M. (2025) https://BioRender.com/myhygrh (accessed on 18 June 2025).

Finally supplanted, platinum-based chemotherapy was the preferred upfront treatment for locally advanced or metastatic urothelial cancer since the 1970s [64]. Combinations such as gemcitabine with either cisplatin or carboplatin and MVAC have historically been the regimens of choice [65,66]. In 2010, FDA approval for immunotherapy maintenance in chemotherapy responders paved the way for more recent advances in the field [67]. Novel therapies for urothelial cancer have changed the treatment landscape within the past few years (Figure 1). Below, we discuss recent advances and pipeline therapeutics for metastatic UC (Table 2).

### 4.1. Chemoimmunotherapy

Similar to the NIAGARA trial in localized MIBC, CheckMate-901 studied combination chemoimmunotherapy in the locally advanced or metastatic setting [68]. This was an open-label phase III trial that randomized cisplatin-eligible patients who had not received prior systemic treatment for advanced or metastatic disease to receive GC with or without nivolumab. Notably, patients with a creatinine clearance ≤ 40 were included in this study. A total of 608 patients were assigned to each group. After a median follow-up of 33.6 months, patients in the nivolumab group demonstrated a significant benefit in progression-free survival (PFS) (7.9 vs. 7.6 months; HR 0.72, *p* = 0.001) and OS (21.7 vs. 18.9 months; HR 0.78, *p* = 0.02).

Building on the recent introduction of LAG-3 inhibitors into the realm of immunotherapy, an active phase I/II substudy under KEYMAKER-U04 (NCT05845814) is investigating the combination of pembrolizumab with the anti-LAG-3 antibody favezelimab in the first line.

### 4.2. The Next Wave of Antibody-Drug Conjugates

#### 4.2.1. Nectin-4

The combination of enfortumab vedotin and pembrolizumab represents what is probably the most significant paradigm shift in the treatment of locally advanced and metastatic urothelial cancer. The landmark phase III EV-302 trial randomized patients with untreated locally advanced or metastatic urothelial cancer to receive either EV + pembrolizumab (EV + P) or platinum-based chemotherapy [27]. This was a large study of 886 patients randomized equally to each arm. At the time of initial publication after a median follow-up of 17.2 months, there was a near doubling of both PFS and OS in the EV + P arm; PFS 12.5 vs. 6.3 months (HR 0.45, *p* < 0.001), OS 31.5 vs. 16.1 months (HR 0.47, *p* < 0.001). As of the updated analysis from February 2025, after an additional year of follow-up, continued survival benefit was observed (OS 33.8 vs. 15.9 months, HR 0.51) [69]. Compared to the chemotherapy arm, EV + P was associated with higher rates of neuropathy (50% vs. 9.9%), alopecia (33.2% vs. 7.9%), and rash (32.7% vs. 3.2%), among other toxicities.

Newer nectin-4 targeting ADCs are also under investigation. The second-generation molecule 9MW2821 has a novel linker that forms site-specific disulfide bonds between the antibody and an MMAE payload, resulting in a homogenous drug–antibody ratio [70]. Preliminary results from a phase I/IIa study of 9MW2821 in pretreated patients with a variety of solid tumors (NCT05216965) showed that among 37 patients with advanced urothelial cancer, an ORR of 62.2% and median PFS of 8.8 months was observed [71]. Phase II (NCT06823427) and III (NCT06592326, NCT06196736) trials of this drug with and without checkpoint inhibitor across lines of therapy are ongoing. A similar compound, SHR-A2102, consisting of a fully humanized IgG1 monoclonal antibody linked to a topoisomerase 1 payload, is being studied in a phase I study (NCT05735275). In a 73-patient cohort of heavily pretreated patients, investigators observed an ORR of 38.4% (32.3% in 6 mg/kg cohort, 50% in 8 mg/kg cohort), with no complete responses. Six-month PFS was 41.4% overall [72]. Finally, NEXUS-01 (NCT06465069), a phase I trial of LY4052031, consisting of an Fc-silent IgG1 antibody conjugated to the novel topoisomerase 1 payload camp98 and EXCEED (NCT06238479), a phase I trial of LY4101174 (ETx-22), a humanized Fc-silent antibody conjugated to MMAE, are both actively accruing patients to study.

#### 4.2.2. HER2

Trastuzumab deruxtecan (T-DXd), an anti-HER2 ADC with a topoisomerase 1 payload, was initially introduced in the breast cancer space but has now gained tumor-agnostic approval in the advanced/metastatic setting based on the phase 2 DESTINY-PanTumor02 trial [73]. In the urothelial cohort of this study, results from which were last updated in May 2024, 41 patients with HER2-expressing (IHC 2+ or 3+) locally advanced or metastatic disease that had progressed on one of more prior lines of treatment received monotherapy T-DXd. The ORR was 39%, and median PFS was 7.0 months.

Disitamab vedotin (DV), another HER2-directed ADC, is also being studied in the advanced/metastatic setting. Two single-arm phase II trials (RC48-C005 and RC48-C009) evaluated DV in patients with advanced HER2-positive urothelial cancer who progressed on at least one prior line of systemic chemotherapy [74]. In a combined analysis of 107 patients in these studies, the ORR was 50.5%, and median PFS and OS were 5.9 and 14.2 months, respectively. Of note, significant adverse effects (any-grade) included peripheral neuropathy (68.2%), cytopenias (50.5% leukopenia and 42.1% leukopenia), alopecia (40.2%), and elevated transaminases (AST 42.1%, ALT 35.5%). This promising data led to other trials in this space. RC48-C014 was a phase Ib/II trial of DV combined with toripalimab in cisplatin-ineligible patients with no restriction on prior treatment [75]. Forty-one patients were enrolled and with a median follow-up of 34.1 months; the median PFS and OS were 9.3 and 33.1 months, respectively, while the ORR was 75%. Similarly, RC48-G001 (NCT04879329) is a multi-cohort phase II study of DV with pembrolizumab in patients with HER2-expressing urothelial tumors (IHC ≥ 1+). In September 2024, data was published for Cohort C, which consists of 20 treatment-naïve patients who received DV plus pembrolizumab followed for a median of nine months. The ORR was 61.1% in all patients (16.7% CR) and 76.9% in HER2-low patients (23.1% CR) [76]. Additional studies are currently enrolling including Cohorts A and B (non-treatment naïve), Part 2 of Cohort C (comparison of DV vs. DV + pembrolizumab), and the phase III DV-001 (DV + pembrolizumab vs. platinum-based chemotherapy in treatment-naïve patients) (NCT05911295) [76]. Finally, the phase III study RC48-C016 (NCT05302284) evaluating first-line DV plus toripalimab against chemotherapy in patients with HER2-expressing locally advanced or metastatic urothelial cancer was announced to have met its dual primary endpoints of PFS and OS in a recent press release.

#### 4.2.3. Trop-2

Datopotamab deruxtecan (Dato-DXd) is a newer ADC composed of a monoclonal antibody to Trop-2 linked to a topoisomerase I inhibitor. TROPION-PanTumor01 (NCT03401385) is an ongoing phase I trial of Dato-DXd in a variety of previously treated advanced solid tumors, including a cohort specific to urothelial cancer. Updated results from this cohort were presented in February 2025 with a median follow-up of 10 months [77]. Forty patients with locally advanced or metastatic urothelial cancer treated with at least one prior line of therapy (including an immune checkpoint inhibitor) received treatment with Dato-DXd monotherapy. The overall response rate was 25% including one CR. The median PFS was 6.9 months to date, and the median duration of response was not reached. Further trials in this space are ongoing, including the closed-to-enrollment phase I/II TROPION-PanTumor02 (NCT05460273) and actively recruiting phase II TROPION-PanTumor03 (NCT05489211) [78].

It is also relevant to include sacituzumab govitecan (SG), another Trop-2-targeting ADC, which as mentioned above no longer has approval for advanced/metastatic urothelial cancer. SG was initially approved based on the phase 2 TROPHY-U-01 trial, but later withdrawn after the phase 3 TROPiCS-04 did not meet its OS endpoint and was notable for increased mortality signals [35]. A related drug, sacituzumab tirumotecan (formerly MK-2780/SKB264), is being studied in the phase I/II MK-2870-001 trial (NCT04152499) in patients with advanced solid tumors who have progressed after standard therapies. This compound contains the novel topoisomerase I inhibitor payload, KL610023, with a hydrolytically cleavable linker permitting both intracellular and extracellular cleavage. The latest results from Cohort 9 evaluating patients with advanced urothelial cancer were reported in February 2025 [79]. Forty-nine patients were treated, 11 as second-line and 38 third-line or later. ORR was 45.5% and 26.3% for these two groups, respectively; however, notably, a significant number of patients had discontinued treatment (mostly due to progression) at the time of data cut-off. Additionally, grade ≥ 3 anemia (38.8%) and decreased neutrophil count (28.6%) were observed. In a related phase I/II trial (NCT06483334), sacituzumab tirumotecan is being studied in combination with EV and pembrolizumab.

Building off the platform provided by these novel drugs, the phase I DAD (Double Antibody Drug Conjugate) trial looked at the combination of SG plus EV [80]. Twenty-three patients with metastatic urothelial cancer who had progressed on platinum and/or immunotherapy received treatment. The ORR was 70% with three complete responses, and at the time of publication after a median follow-up of 14 months, nine patients had ongoing responses. Grade ≥3 adverse events were observed in 78% of patients, including anemia (34.8%), decreased neutrophil count (17.4%), urinary tract infection (13.0%), and one case of grade 5 pneumonitis. An additional cohort (DAD-IO) is now enrolling to combine SG and EV with pembrolizumab (NCT04724018).

#### 4.2.4. Other Novel Targets

Lastly, the most recent advance in this class has been the development of the bispecific ADC. BL-B01D1 is a first-in-class ADC comprised of an EGFR-HER3 bispecific antibody linked to a novel topoisomerase I inhibitor (Ed-04). The results of a phase Ib/II trial published in September 2024 showed that in a small cohort of 32 patients with previously treated advanced or metastatic urothelial cancer, ORR was 43.5% and median PFS was 5.5 months [81]. Of note, there was no restriction on HER2 expression in this study. Grade ≥3 adverse events included anemia (17%), leukopenia (26%), thrombocytopenia (26%), and neutropenia (22%). Further phase II (NCT05785039) and III (NCT06857175) trials are now recruiting, testing this drug as monotherapy, as well as a phase II trial in combination with PD-1 inhibitor (NCT06405425).

### 4.3. Small Molecule–Drug Conjugates

Zelenectide pevedotin (formerly BT8009), a bicyclic peptide–drug conjugate targeting nectin-4 with a linker to MMAE, is a member of a novel class of compounds being investigated in advanced urothelial cancer. The phase I/II Duravelo-1 trial (NCT04561362) is evaluating this drug as monotherapy and in combination with pembrolizumab in patients who had progressed on checkpoint inhibitor and platinum-based chemotherapy (or were ineligible). In the latest update from September 2024, ORR was 45% among 38 patients who received zelenectide pevedotin as monotherapy [82]. The phase II/III study, Duravelo-2 (NCT06225596), is now recruiting for two cohorts based on prior treatment [83]. Cohort 1 randomizes previously untreated patients eligible for platinum-based chemotherapy to BT8009 monotherapy, BT8009 in combination with pembrolizumab, or gemcitabine plus cisplatin/carboplatin followed by avelumab maintenance. Cohort 2 randomizes previously treated patients to different doses of BT80009, with plans to open a combination arm with pembrolizumab.

BT5528 is another bicyclic conjugate that targets EphA2, a tumor antigen expressed in many tumors. In the phase I/II study that included 128 patients (25% urothelial cancer), the highest antitumor activity was seen in the urothelial cohort, with ORR of 45% and 27% (confirmed and unconfirmed responses, respectively) in evaluable patients [84]. The study is still recruiting for expansion cohorts and is also investigating BT5528 in combination with nivolumab (NCT04180371).

Finally, BT7480 is comprised of three bicyclic peptides, one targeting nectin-4 and two targeting CD137 (4-1BB) on immune cells. A phase I/II study (NCT05163041) is evaluating this drug as monotherapy and in combination with nivolumab. Initial results from 39 patients with a variety of nectin-4-expressing solid tumors reported three patients with prolonged stable disease, but this was not specific to urothelial cancer [85].

### 4.4. Targeted Therapies in Advanced/Metastatsic Urothelial Cancer

Fibroblast growth factor receptors (FGFR) are involved in the regulation of angiogenesis as well as cell proliferation, migration, and survival. Alterations in FGFR2 and FGFR3 in particular have been identified as frequent drivers in the development of bladder cancers [86,87]. The first major breakthrough in this space came with the approval of the pan-FGFR inhibitor erdafitinib as monotherapy in the treatment-refractory setting based on the results of the phase II THOR study [88]. In this study, patients with FGFR2/3 alterations were randomized to either chemotherapy or erdafitinb, with the latter demonstrating survival benefit (OS 12.1 vs. 8.9 months; HR 0.64, *p* = 0.005). Of note, erdafitinib was associated with significant toxicities including grade ≥ 3 palmar–plantar erythrodysesthesia syndrome (9.6%) and hyperphosphatemia (5.2%). Further trials are attempting to build on this drug and other related compounds. The phase II NORSE trial randomized treatment-naïve cisplatin-ineligible patients with susceptible FGFR-altered tumors to receive either erdafitinib alone or with cetrelimab [89]. Final results published in May 2023 reported, among 87 patients, an ORR rate of 54.5% (13.6% CR) in the combination arm and 44.2% (2.3% CR) in the erdafitinib monotherapy arm. The median PFS was 11.0 vs. 5.6 months, favoring the combination arm. A related phase II trial studied the combination of the novel FGFR inhibitor futibatinib plus pembrolizumab in 43 treatment-naïve patients with cisplatin-ineligible tumors with or without FGFR alterations [90]. In the cohort of 17 patients with FGFR mutations, ORR was 47% (12% CR) with a median PFS of 8.3 months and a 12-month OS of 62%. The 26-patient cohort without FGFR alterations had an ORR of 31% (12% CR) with a median PFS of 4.8 months and a 12-month OS of 57%.

Next-generation FGFR inhibitors that selectively target FGFR3 are also currently in clinical trials, as it is believed that off-target inhibition of the FGFR 1/2/4 drives some of the toxicity seen with pan-inhibitors. LOXO-435 is being studied in the phase I FORAGER-1 trial (NCT05614739) of FGFR3-altered advanced solid tumors that progressed after standard therapies [91]. As of initial results published in February 2025, 101 patients were enrolled, 70% of which had urothelial cancer. After a median follow-up of five months in the urothelial cohort, ORR was 42% (and 45% in patients who had previously received an FGFR inhibitor). Treatment-emergent adverse events associated with poor tolerance and compliance to erdafitinib were reported as low-grade and with <5% incidence; however, diarrhea and hyperphosphatemia were still common. Similarly, the selective FGFR3 inhibitor TYRA-300 is being studied in the currently enrolling phase I/II SURF301 trial (NCT05544552) [92].

Finally, FGFR inhibitors are also being investigated in combination with other therapies. ETCTN 10483 (NCT04963153) is a phase I/II trial of patients with advanced urothelial carcinoma and FGFR3/2 gene alterations who have progressed after chemotherapy and/or immunotherapy, in which patients received erdafitinib with EV. Results from nine patients in the dose-escalation phase were published in February 2025 after a median follow-up of 22.7 months [93]. The ORR was 100% (11.1% CR); median PFS was 7.5 months and median OS was not reached.

### 4.5. Cellular Therapies

Combining two well-established immunotherapy targets, SI-B003 is a dual-checkpoint bispecific antibody targeting both PD-1 and CTLA-4. A phase I trial (NCT04606472) is investigating this therapy in a variety of advanced solid tumors progressed after standard therapies [94]. Data has not been released for the urothelial-specific cohort at this time. Building on this study, a phase II trial (NCT05965856) is underway exploring SI-B003 as monotherapy and in combination with BL-B01D1, the previously mentioned EGFR-HER3 bispecific antibody, with a focus on patients with advanced urothelial carcinoma.

DF1001 is a first-in-class tri-specific NK cell engager targeting HER2 that is being tested in an enrolling phase I/II clinical trial of advanced and/or refractory solid tumors. There are specific cohorts for patients with urothelial cancer, consisting of either DF1001 monotherapy or in combination with other agents (NCT04143711) [95].

Lastly, the phase I VISTA trial (NCT03740256) combines an immunomodulatory oncovirus with a novel CAR-T therapy in HER2-positive disease [96]. CAdVEC, a binary oncolytic adenovirus with dual expression of IL-12 and PD-L1 inhibitor, will be used in conjunction with a HER2-sepcific CAR-T product for advanced HER2-postiive (IHC ≥ 2+) solid tumors who have progressed after standard treatments.

## 5. Conclusions and Future Directions

The treatment of both localized and advanced urothelial cancer has changed drastically in recent years. What was once a disease with a dismal prognosis, a high attrition rate after first line treatment, and limited treatment options has become a condition that we look upon with optimism. For early-stage bladder cancers where cystectomy is still an integral part of the treatment algorithm, bladder-sparing strategies for eligible patients are enthusiastically being explored.

The standard of care for MIBC at the time of writing remains neoadjuvant treatment followed by radical cystectomy. For those who are cisplatin-eligible, the current evidence supports neoadjuvant cisplatin-based chemotherapy with perioperative immunotherapy, based mainly on the NIAGARA trial [14]. Conversely, such evidence does not exist for the cisplatin-ineligible population, representing a significant population with an unmet need. Strategies under investigation generally include either neoadjuvant or perioperative treatment with some combination of chemotherapy, immunotherapy, radiation, and/or novel therapies. Enfortumab vedotin, sacituzumab govitecan, and targeted agents based on tumor-specific molecular profiles are all being evaluated in this space. Newer approaches such as intravesical treatments and personalized vaccines have also shown promise.

However, these consensus and investigational treatment options all still include radical cystectomy as a core component. Bladder-sparing approaches have the potential to revolutionize the field. Such approaches can include purely systemic treatments or multidisciplinary trimodality therapy. In the former, patients receive combination chemoimmunotherapy, after which if they display evidence of complete response on post-treatment TURBT, they may defer cystectomy and enter surveillance with or without concomitant maintenance immunotherapy. On the other hand, trimodality therapy, which carries a category 1 recommendation, has also been investigated with newer therapies. While conventional trimodality therapy includes maximal TURBT followed by concurrent chemoradiation, novel approaches consist of either immunotherapy or ADCs in addition to various radiotherapy regimens. Both bladder-sparing strategies have shown promising rates of pCR on post-treatment TURBTs, resulting in many patients retaining their bladders without late recurrences.

Considering locally advanced or metastatic disease, the landmark EV-302 trial established the combination of enfortumab vedotin and pembrolizumab as the gold-standard first-line option [27]. This regimen has the benefit of being independent of renal function, so cisplatin eligibility no longer applies to this situation and allows more patients to benefit from treatment. For those patients with contraindications to this regimen (which may include autoimmune disease, significant peripheral neuropathy, or severe skin conditions), conventional chemotherapy options remain viable.

Building off the success of immunotherapy and ADCs, there is a plethora of new and exciting therapies being tested first in the locally advanced and metastatic setting. Newer immunotherapies, including anti-LAG-3 antibodies, are being added to standard regimens. Next-generation ADCs targeting nectin-4, HER-2, and Trop-2, as well as a bispecific ADC targeting EGFR and HER3, are all under investigation. A new class of compounds comprised of small molecule–drug conjugates have been created with various targets, including nectin-4. Newer FGFR inhibitors, both selective and non-selective, are being studied alone and in combination with other agents. Finally, cellular therapies such as immune-targeting bispecific antibodies, trispecific NK cell engagers, and CAR-T therapies are in early-stage clinical trials.

With the introduction of new and targeted treatment modalities as well as advanced testing options, molecular analysis looks to be promising for the field. Circulating tumor DNA has emerged as a potential marker for predicting prognosis and clinical response, particularly in the adjuvant setting. Several ongoing trials attempt to use ctDNA as a tool to identify which patients might be at higher risk for relapse and thus warrant intensification of treatment following radical cystectomy. Molecular profiling of tumors can also identify biomarkers that might portend benefit to certain targeted treatments.

In conclusion, the last few years have been ripe with major advances in the treatment of urothelial cancer. We now have at our disposal very effective regimens for both early-stage and advanced disease. Ongoing trials hope to build on the current landscape by instituting bladder-sparing strategies for MIBC and novel systemic therapies in advanced patients.

## Figures and Tables

**Table 1:** Select recent and ongoing clinical trials for MIBC.

Trial Number	Investigational Drug	Mechanism	Cisplatin Eligibility	Trial Status	Location	Phase
NCT04876313	Neoadjuvant Nab-paclitaxel + perioperative Nivolumab	Anti-PD-1	Ineligible	Recruiting	Europe	2
NCT05241340	Neoadjuvant Sasanlimab + concurrent SBRT	Anti-PD-1	Ineligible	Recruiting	U.S.	2
NCT04813107	Neoadjuvant Tislelizumab +/− APL-1202	Anti-PD-1, MetAP2 inhibitor	Ineligible	Unknown	Global	2
NCT06571708	Gemcitabine/Cisplatin with Cemiplimab +/− Fianlimab	Anti-PD-1, Anti-LAG-3	Eligible	Not yet recruiting	U.S.	2
NCT05987241	Adjuvant Nivolumab +/− Relatlimab	Anti-PD-1, Anti-LAG-3	N/A	Recruiting	U.S.	2/3
NCT03924856	Perioperative Pembrolizumab (with neoadjuvant chemotherapy)	Anti-PD-1	Eligible	Active, not recruiting	Global	3
NCT02632409	Adjuvant Nivolumab	Anti-PD-1	N/A	Active, not recruiting	Global	3
NCT03661320	Perioperative Nivolumab (with neoadjuvant chemotherapy) +/− Linrodostat	Anti-PD-1, IDO1 inhibitor	Eligible	Active, not recruiting	Global	3
NCT04209114	Perioperative Nivolumab +/− Bempeg (NKTR-214)	Anti-PD-1, IL-2 prodrug	Ineligible	Completed	Global	3
NCT03732677	Perioperative Durvalumab (with neoadjuvant chemotherapy)	Anti-PD-L1	Eligible	Active, not recruiting	Global	3
NCT03288545	Enfortumab Vedotin (various settings)	Nectin-4 ADC	Ineligible	Active, not recruiting	Global	1b/2
NCT06394570	Neoadjuvant Enfortumab Vedotin + SBRT	Nectin-4 ADC	Ineligible	Recruiting	U.S.	1/2
NCT03924895	Perioperative Enfortumab Vedotin +/− Pembrolizumab	Nectin-4 ADC, Anti-PD-1	Ineligible	Active, not recruiting	Global	3
NCT04700124	Perioperative Enfortumab Vedotin + Pembrolizumab	Nectin-4 ADC, Anti-PD-1	Eligible	Active, not recruiting	Global	3
NCT04960709	Neoadjuvant Enfortumab Vedotin with Durvalumab +/− Tremelimumab	Nectin-4 ADC, Anti-PD-L1, Anti-CTLA-4	Ineligible	Active, not recruiting	Global	3
NCT05226117	Neoadjuvant Sacituzumab Govitecan	Trop-2 ADC	Ineligible	Unknown	Europe	2
NCT05535218	Neoadjuvant Sacituzumab Govitecan + perioperative Pembrolizumab	Trop-2 ADC, Anti-PD-1	Ineligible	Active, not recruiting	Europe	2
NCT05297552	Neoadjuvant Disitamab Vedotin + Toripalimab	HER2 ADC, Anti-PD-1	Ineligible	Recruiting	Asia	2
NCT06511648	Neoadjuvant Erdafitinib +/− Cetrelimab	FGFR inhibitor, Anti-PD-1	Ineligible	Recruiting	Europe	2
NCT04919512	Neoadjuvant Cetrelimab +/− TAR-200 gemcitabine	Anti-PD-1, intravesical gemictabine delivery system	Ineligible	Active, not recruiting	Global	2
NCT04610671	Neoadjuvant GC0070 (Cremostigene) + Nivolumab	Oncolytic adenovirus, Anti-PD-1	Ineligible	Active, not recruiting	U.S.	1b
NCT03359239	Adjuvant PGV001 + Atezolizumab	Multi-peptide neoantigen vaccine, Anti-PD-1	N/A	Completed	U.S.	1
NCT06305767	Perioperative V940 + Pembrolizumab and Enfortumab Vedotin (phase 1), Adjuvant Pembrolizumab +/− V940 (phase 2)	mRNA vaccine, Anti-PD-1, Nectin-4 ADC	Ineligible	Recruiting	Global	1/2
NCT06534983	Adjuvant Autogene Cevumeran +/− Nivolumab	mRNA vaccine, Anti-PD-1	Ineligible	Recruiting	Global	2

**Table 2:** Recent and ongoing trials for advanced/metastatic bladder cancer.

NCT	Investigational Drug	Mechanism	Line of Therapy	Trial Status	Location	Phase
NCT05845814	Pembrolizumab + Favezelimab	Anti-PD-1, Anti-LAG-3	First-line	Active, not recruiting	Global	1/2
NCT03036098	Ipilimumab/Nivolumab (Arm A), Gemcitabine/Cisplatin +/− nivolumab (Arm C)	Anti-PD-1, Anti-CTLA-4	First-line	Active, not recruiting	Global	3
NCT05216965	9MW2821	Nectin-4 ADC	Unspecified	Recruiting	Asia	1a/2
NCT05735275	SHR-A2102	Nectin-4 ADC	Unspecified	Recruiting	Asia	1
NCT06465069	LY4052031	Nectin-4 ADC	Refractory	Suspended	Global	1
NCT06238479	LY4101174	Nectin-4 ADC	Refractory	Recruiting	Global	1
NCT06196736	9MW2821 + Toripalimab	Nectin-4 ADC, Anti-PD-1	Refractory	Recruiting	Asia	3
NCT06823427	9MW2821 +/− Toripalimab	Nectin-4 ADC, Anti-PD-1	First-line	Recruiting	Asia	2
NCT06592326	9MW2821 + Toripalimab	Nectin-4 ADC, Anti-PD-1	First-line	Recruiting	Asia	3
NCT04223856	Enfortumab Vedotin + Pembrolizumab	Nectin-4 ADC, Anti-PD-1	First-line	Active, not recruiting	Global	3
NCT04264936	Disitamab Vedotin + Toripalimab	HER2 ADC, Anti-PD-1	Refractory	Unknown	Asia	1b/2
NCT04482309	Trastuzumab Deruxtecan	HER2 ADC	Refractory	Recruiting	Global	2
NCT04879329	Distiamab Vedotin + Pembrolizumab	HER2 ADC, Anti-PD-1	Any	Recruiting	Global	2
NCT05911295	Disitamab Vedotin + Pembrolizumab	HER2 ADC, Anti-PD-1	First-line	Recruiting	Global	3
NCT05302284	Disitamab Vedotin + Toripalimab	HER2 ADC, Anti-PD-1	First-line	Recruiting	Asia	3
NCT05460273	Datopotamab Deruxtecan	Trop-2 ADC	Unspecified	Active, not recruiting	Asia	1/2
NCT05489211	Datopotamab Deruxtecan +/− other agents	Trop-2 ADC	Unspecified	Recruiting	Global	2
NCT03401385	Datopotamab Deruxtecan	Trop-2 ADC	Refractory	Active, not recruiting	Global	1
NCT04152499	Sacituzumab Tirumotecan	Trop-2 ADC	Refractory	Recruiting	Global	1/2
NCT06483334	Sacituzumab Tirumotecan + Enfortumab Vedotin +/− Pembrolizumab	Trop-2 ADC, Nectin-4 ADC, Anti-PD-1	Any	Recruiting	Global	1/2
NCT05785039	BL-B01D1	Bispecific EGFR-HER3 ADC	Refractory	Recruiting	Asia	2
NCT06857175	BL-B01D1	Bispecific EGFR-HER3 ADC	Refractory	Recruiting	Asia	3
NCT06405425	BL-B01D1 + Anti-PD-1 (unspecified)	Bispecific EGFR-HER3 ADC, Anti-PD-1	First-line	Recruiting	Asia	2
NCT04561362	Zelenectide Pevedotin +/− Pembrolizumab	Nectin-4 toxin conjugate, Anti-PD-1	Refractory	Recruiting	Global	1/2
NCT06225596	Zelenectide Pevedotin +/− Pembrolizumab	Nectin-4 toxin conjugate, Anti-PD-2	Any	Recruiting	Global	2/3
NCT04180371	BT5528 +/− Nivolumab	EphA2 toxin conjugate, Anti-PD-1	Refractory	Recruiting	Global	1/2
NCT05163041	BT7480 +/− Nivolumab	Nectin-4/CD137 toxin conjugate, Anti-PD-1	Refractory	Active, not recruiting	Global	1/2
NCT05544552	TYRA-300	FGFR3 inhibitor	Refractory	Recruiting	Global	1/2
NCT04963153	Erdafitinib + Enfortumab Vedotin	Pan-FGFR inhibitor, Nectin-4 ADC	Refractory	Recruiting	Global	1/2
NCT03390504	Erdafitinib	Pan-FGFR inhibitor	Refractory	Active, not recruiting	Global	3
NCT05614739	LOXO-435 +/− Pembrolizumab +/− Enfortumab Vedotin	FGFR3 inhibitor, Anti-PD-1, Nectin-4 ADC	Any	Recruiting	Global	1
NCT03473743	Erdafintib + either platinum-based chemotherapy or Cetrelimab	Pan-FGFR inhibitor, Anti-PD-1	Any	Active, not recruiting	Global	2
NCT04601857	Fudibatinib + Pembrolizumab	Pan-FGFR inhibitor	First-line	Active, not recruiting	Global	2
NCT04606472	SI-B003	PD-1/CTLA-4 bispecific antibody	Refractory	Recruiting	Asia	1
NCT05965856	SI-B003 +/− BL-B01D1	PD-1/CTLA-4 bispecific antibody, Bispecific EGFR-HER3 ADC	Refractory	Recruiting	Asia	2
NCT04143711	DF1001 +/− Nivolumab, chemotherapy or Sacitizumab Govitecan	Trispecific HER2 NK cell engager, Anti-PD-1	Refractory	Recruiting	Global	1/2
NCT03740256	CAdVEC + HER2-directed CAR-T	Oncolytic adenovirus, CAR-T	Refractory	Recruiting	U.S.	1
NCT03359239	PGV001 + Atezolizumab	Multi-peptide neoantigen vaccine, Anti-PD-1	Refractory or switch-maintenance	Completed	U.S.	1
